# A comparison of health state utility values associated with oral potentially malignant disorders and oral cancer in Sri Lanka assessed using the EQ-5D-3 L and the EORTC-8D

**DOI:** 10.1186/s12955-016-0502-y

**Published:** 2016-07-11

**Authors:** Sanjeewa Kularatna, Jennifer A. Whitty, Newell W. Johnson, Ruwan Jayasinghe, Paul A. Scuffham

**Affiliations:** Centre for Applied Health Economics, School of Medicine, Nathan Campus, Griffith University , 170 Kessels Road, Nathan, 4111 Queensland Australia; Menzies Health Institute Queensland, Griffith University, Queensland, Australia; School of Pharmacy, The University of Queensland, Queensland, Australia; Faculty of Dental Sciences, University of Peradeniya, Peradeniya, Sri Lanka

## Abstract

**Background:**

It has been suggested that the EQ-5D-3 L preference-based measure of health outcome lacks sensitivity to discriminate between health states in cancer patients. An alternative approach is to use a disease (cancer) specific preference-based measure, such as the EORTC-8D. A limited number of comparisons have been made between generic and disease specific preference-based measures. The aim of this study was to compare the utility scores from the EQ-5D-3 L and the EORTC-8D in a group of patients with oral cancer or with oral potentially malignant disorders (OPMD).

**Methods:**

Patients (*n* = 151) with OPMD or oral cancer were recruited consecutively from six hospitals in Sri Lanka. All participants completed both the EQ-5D-3 L and the EORTC's QLQC-30 instrument. The Sri Lankan EQ-5D-3 L and EORTC-8D scoring algorithms were employed to estimate utility scores. The utility scores from the two instruments were compared for discrimination, responsiveness and correlation.

**Results:**

There were significant differences across the two utility scores. The EQ-5D-3 L showed better discrimination than EORTC-8D with higher effect sizes. There were higher ceiling effects observed in the EQ-5D-3 L. There was poor correlation between the dimensions of the two instruments except for the mobility and physical functions.

**Conclusion:**

The two instruments captured different aspects of quality of life. The EQ-5D-3 L demonstrated better discrimination than the EORTC-8D. In mild conditions EORTC-8D was more responsive and we recommend further validation of this instrument in diverse cancer conditions.

## Background

Economic evaluation is an important aspect of resource allocation in health care. Cost utility analysis (CUA) is the most popular method of evaluation in contemporary health economics [1]. CUA uses Quality Adjusted Life Years (QALYs) along with costs as the principle outcome measures. The use of QALYs as an outcome measure permits evaluation of benefits across different interventions and diseases where QALYs capture changes in both quantity and quality of life. The QALY plays a major role in deciding whether a new treatment provides relative benefits for any additional cost. Thus, this outcome measure and its sensitivity to discriminate quality of life changes are crucial in determining value for money.

QALYs use utility scores to quantify the preference for a given health state [2]. Multi attribute utility instruments (MAUIs), such as the EQ-5D-3 L, have facilitated health state valuation, with utility scores assigned to all health states described by the MAUI based on public preferences for being in that health state [3, 4]. However, with the advent of a number of MAUIs [3–7], it is unclear which instrument performs best in different disease states [8]. Currently, the National Institute for Health and Care Excellence (NICE) in the United Kingdom recommends the EQ-5D-3 L for economic evaluations [2]. However, it is unclear whether this instrument is capable of discerning utility values in complex disease conditions that have multiple physical, social, psychological and economic complications [9]. The recent development of EQ-5D-5 L is considered to improve the crude three level structure of the EQ-5D-3 L by having 3125 health states. However, national level health state valuations and comparisons should be conducted in several countries to ascertain the true nature of the improvement.

Cancer is a disease condition where debate continues as to whether disease-specific preference-based measures or generic measures perform better [9, 10]. It is also a frontier of research into new treatments aiming to improve both longevity and quality of life, so it is important that the best available utility instruments are employed. Recently, a preference-based measure, the EORTC-8D, was developed using the 30 items in EORTCs’ QLQC-30 instrument for use as a disease (cancer) specific preference based measure [9]. An algorithm developed in a health state valuation study facilitates estimating EORTC-8D utility values directly from the QLQC-30 data [9]. Therefore, in clinical trials and research, one instrument (the QLQ-C30) can be used to measure changes in quality of life as well as the utility of cancer health states, reducing the burden of data collection. For utilities produced with measures like EORTC-8D, it is important to test whether they are really more discriminative and responsive than a generic instrument, such as the EQ-5D-3 L, despite their larger number of potential health states. Whether the utility differences produced from the disease specific instrument are significantly different from generic utility scores between severity groups and over time also needs to be examined. Furthermore, it is important to ascertain whether the health spaces covered by the disease specific instrument are different to the coverage by generic instruments: Few studies have addressed this [10, 11]. There is little comparative information between the EQ-5D-3 L and disease specific instruments and a gap in the literature about the construct validity of preference based disease specific instruments.

One of the major cancer conditions experienced in Sri Lanka is oral cancer. There are current interests to determine the cost-effectiveness of oral cancer screening and different treatment methods in Sri Lanka. Therefore, it is timely to determine which preference-based measure is most sensitive to the health states associated with this malignancy, for use in future cost utility analyses. When considering oral cancer, precursor lesions, termed Oral Potentially Malignant Disorders (OPMD) [12], are also important. Major classifications of OPMD, namely Leukoplakia, Erythroplakia and Oral submucous fibrosis, are associated with a risk of malignant transformation [13, 14]. The term oral cancer includes malignancies of the lip, tongue, buccal mucosa, gingiva and other tissues within the mouth. Cancers of the lip, oral cavity and oropharynx, taken together, are the most prevalent cancers among Sri Lankan males with an age standardized incidence rate of 15.4 per 100, 000 population per annum [15]. Oral cancer in Sri Lanka carries the highest mortality rate among all cancers of 7.1 per 100,000 pa. [16]. Oral cancer has a substantial impact on quality of life (QoL), particularly through associated disfigurement, speech, eating and swallowing difficulties, drooling, financial and social constraints, emotional disturbances, and issues related to oral rehabilitation and depression/anxiety due to fear of recurrence. People presenting with late stage cancer and patients who have received combined surgery and radiotherapy as treatment are more likely to have severe reductions in their QoL compared with those who receive surgery alone for smaller lesions [17].

The aim of this study is to ascertain if there are significant differences between the utility values estimated from the EQ-5D-3 L and the EORTC-8D for the same OPMD and oral cancer health states. The study also sought to determine the discriminative ability of the two preference measures for OPMD and oral cancer health states. Findings will have important implications for the selection of MAUIs to value quality of life in studies and clinical practice related to the management or oral health states.

## Methods

A cross sectional study design was used. Data were collected from OPMD and oral cancer patients from five general hospitals from five districts of the country plus a Dental Teaching Hospital in a sixth district (Kandy) in Sri Lanka. Inclusion of six districts ensured diversity of the sample. During the months of June 2013 to February 2014 consecutive individuals diagnosed with an OPMD or with oral cancer attending the Maxillo-facial clinics and oral surgery wards were invited to take part. All oral cancer patients who consented to take part in the study and attended regular review clinics during this time period were also recruited. However, OPMD patients were recruited only at diagnosis: those on review visits were excluded as most of the review OPMD patients who had undergone treatment do not have quality of life problems associated with the disease. Oral cancer patients just diagnosed (prior to surgery), after surgery, during and after adjunctive chemotherapy/radiotherapy and who came for review visits were included. Ethical clearance was obtained from Griffith University Human Research Ethics Committee (MED/29/12/HREC) and the Sri Lanka Medical Association (ERC/12/022).

### Study instruments

Both EQ-5D-3 L and the QLQ-C30 were administered to participants at the time of the recruitment. The EQ-5D-3 L is a generic MAUI with five dimensions (mobility, personal care, usual activities, pain/discomfort and anxiety/depression) [3]. Each dimension is classified into three levels (no problem, some problem and severe problems). Therefore, the EQ-5D-3 L can describe the health space with 243 health states. The state of full health (11111) is given the utility value of 1 and that of death, 0. Utility values can be less than zero for health states regarded as worse than death. The QLQ-C30 is a 30 item disease-specific Likert-type questionnaire used widely in cancer research to collect information on QoL [18]. Of the 30 items, 28 measure responses using a four point Likert scale. There are two global health questions with a seven point Likert scale. These 30 items can be scaled to five functional scales, three symptom scales, a global health status and six single items to represent QoL [19]. A higher score for the functional scale or global health status represents better QoL, while a higher score for a symptom scale represents lower QoL. However, the scores for these 30 items cannot be used to develop an algorithm to estimate utilities as the items do not include any preference measure. The EORTC-8D was developed specifically to provide scores for various domains of the QLQ-C30. These scores were derived from preferences of the general population of the UK (n = 350) who are above 18 years of age to avoid being in these health states. The EORTC-8D has eight dimensions (physical functioning, role functioning, pain, emotional functioning, social functioning, fatigue/ sleep disturbance, nausea and constipation /diarrhoea) with four or five levels in each, describing 81,290 health states [9].

### Interview

The data were collected by trained house officers and clinicians attached to the Maxillofacial units. Patients who provided informed consent were interviewed and clinical data captured from their hospital records. This included basic demographic information, co-morbidities and clinical history of the current OPMD or the oral cancer condition of the patient. Subsequently, participants described their current state of health according to both the five domain descriptive system of the EQ-5D-3 L questionnaire and the visual analogue scale (VAS) [20]. Lastly, participants completed the 30-item QLQC-30 questionnaire [19]. All instruments were interviewer administered.

### Analysis

The oral cancer patients were categorised as treated and untreated: the former being patients were who had undergone surgery to remove the tumours (whether or not there was subsequent chemo- or radio-therapy): the latter were waiting for surgery.

#### EORTC QLQ-C30 scaling

Using the recorded QLQC-30 scoring values and the coding syntax from the EORTC, 30 items of the this instrument were converted to one global health status, five functional scales and nine symptom scales [19]. Each of the QLQC-30 scales range from 0–100. A high score in functional and global health status represent higher functioning and better QoL. Higher scores on the symptom scale represent severe problems.

#### Utility scores

Using the scored EQ-5D-3 L questionnaires and the Sri Lankan EQ-5D-3 L utility scoring algorithm, the EQ-5D-3 L utility scores for each patient’s health state were generated [19]. The Sri Lankan EORTC-8D algorithm was used to convert the QLQ C-30 responses to EORTC-8D utility values [9, 21]. Distributions for the EQ-5D-3 L and EORTC-8D were plotted using the mean, standard deviation and the range of the two utility scores.

### Psychometric properties of the two measures

Methods of comparing MAUIs are ambiguous. Rowen et al. compared MAUIs using discrimination, responsiveness, agreement and difference across MAUIs in a recent paper [10]. We followed the same methodology to compare EQ-5D-3 L and EORTC-8D in this group of oral cancer and OPMD patients.

#### Discrimination

A MAUI should be able to distinguish between clinical severity groups. Utility for one severity group (e.g., oral cancer stage) should be different from another. When the severity of the oral cancer increases, utility scores should decrease, reflecting a worse health state. This also provides an element of external validity for the MAUI. The most mild severity group is the OPMD. The more severe clinical groups are oral cancer stages, defined as, Stage I - the cancer is less than 2 cm in size, and has not spread to lymph nodes in the area; Stage 2 - the cancer is more than 2 cm in size, but less than 4 cm, and has not spread to lymph nodes in the area; Stage 3 - the cancer is bigger than 4 cm but has not spread to any lymph nodes or other parts of the body; Stage 4- the cancer is bigger than 4 cm without spread or is any size and has metastasised to regional lymph nodes [22, 23].

Discrimination properties of EQ-5D-3 L and EORTC-8D were explored using statistical and psychometric tests. Kruskal-Wallis test was used to test the significance of difference between severity classes for utilities. The effect size, calculated as the difference in mean utility between two adjacent severity classes divided by the mean utility of the milder severity class, was used to estimate the size of the difference observed between the mean utilities for severity classes. In each severity class, the effect sizes were compared between the EQ-5D-3 L and the EORTC-8D. To compare the effect sizes of the EQ-5D-3 L and the EORTC-8D, larger effect sizes denote better disease discriminating ability [24]. The effect size was categorized into small (0.2-0.5), medium (0.5-0.8) and large (>0.8), and an effect size of 1 indicates change in size similar to one standard deviation [25].

#### Responsiveness

Responsiveness of a MAUI depends on the ability to capture utility change between time points. As this study did not have longitudinal data we were unable to analyse responsiveness. However, another way to categorise responsiveness of an instrument is to examine its ceiling and floor effects [26]. The percentage of “full health” reported for a MAUI determines the ceiling effects. Floor effects are determined by the percentage of patients reporting worst health level in each dimensions [26]. Lower ceiling and floor effects are indicative of good responsiveness for an instrument. Thus, we also compared the two MAUIs for their ceiling and floor effects.

#### Agreement

Due to a potentially skewed distribution of both the EQ-5D-3 L and the EORTC-8D health states [10], Spearman’s rho was used to assess the non-parametric correlation coefficient between the two utility scores and the dimensions between the two instruments [24]. A Spearman’s rho of >0.5 or < −0.5, was considered a strong correlation [25].

#### Difference across MAUIs

It is important to determine whether there is significant difference between EQ-5D-3 L and EORTC-8D utility values for the same health state experienced by patients. The differences were calculated and correlations for each dimension of the two MAUIs examined. Wilcoxon signed rank test was used to determine significant difference between the two utility scores (*p* < 0.05).

## Results

### Demographics and quality of life

There were 151 OPMD and oral cancer patients who gave consent to the study. As the questionnaires were interviewer administered, the majority of patients answered all questions. Three patients who did not provide the QLQC-30 information were excluded from analysis. Table 1 summarises the characteristics of the sample. The majority were males (54.3 %) and above 60 years of age (52.9 %). There were 57 (37.8 %) OPMD patients, predominantly oral lichen planus and oral sub mucous fibrosis; the remainder had oral squamous cell carcinoma. Of the 94 oral cancer patients, 23.4 % were awaiting treatment.

**Table 1:** Demographic characteristics of the sample

Variable	N (%)
Gender	
Male	82(54.3)
Female	69(45.7)
Age group	
18-29	3(2)
30-39	14(9.3)
40-49	22(14.6)
50-59	32(21.2)
60-69	44(29.1)
70 above	36(23.8)
Marital status	
Never married	9(6)
Married	135(89.4)
Widow/separated	7(4.6)
Ethnicity	
Sinhalese	120(79.5)
Tamil	25(16.6)
Muslim	6(4)
Education	
No formal education	16(10.7)
Primary	84(56)
Secondary	44(29.3)
Tertiary	6(4)
Employment	
Employed	105(70)
Non-economic activities	38(25.3)
Family worker	7(4.7)
OPMD/ cancer	
OPMD	57(37.8)
Oral cancer	94(62.2)
OPMD diagnosis *n* = 44	
Leukoplakia	1(2.3)
Mixed red and white lesions	2(4.5)
Oral sub mucous fibrosis	13(29.5)
Lichen planus	19(43.2)
Other	9(20)
Cancer stage *n* = 94	
Oral cancer Stage I	24(29.6)
Oral cancer Stage II	26(32.1)
Oral cancer Stage III	19(23.5)
Oral cancer Stage IV	12(14.8)
Treatment for oral cancer patients *n* = 94	
Treated (post-surgery) *n* = 72	72(76.6)
Awaiting treatment (pre-surgery) *n* = 22	22(23.4)

OPMD- Oral potentially malignant disorder; Stage I -The cancer is less than 2 cm in size, and has not spread to lymph nodes in the area; Stage 2 - The cancer is more than 2 cm in size, but less than 4 cm, and has not spread to lymph nodes in the area; Stage 3 - The cancer is bigger than 4 cm but has not spread to any lymph nodes or other parts of the body; Stage 4- the cancer is either greater than 4 cm in largest diameter or is of any size but has metastasised to regional lymph nodes [22, 23]

### EORTC QLQ-C30 scaling

Table 2 shows the mean scores for the QLQC-30 scales on the quality of life of the patients. The quality of life of oral cancer patients was substantially lower than for OPMD patients across all scales. Patients who had already received treatment for oral cancer had better quality of life than those awaiting treatment. The functional scale mean values and the global health status mean values decreased with increasing stage of disease. The VAS scores were similar to the global health status scores and were lower than the mean values of the functional scales.

**Table 2:** Quality of life of the sample according to severity using QLQC-30

	Full sample Mean(SD)	OPMD	All Oral cancer	Oral cancer Stage I	Oral cancer Stage II	Oral cancer Stage III	Oral cancer Stage IV	Oral cancer treated	Oral cancer awaiting treatment
VAS	66(22)	70(22)	63(20)	68(17)	62(19)	57(23)	53(14)	67(22)	57(17)
GHS	61(23)	68(23)	57(23)	64(15)	57(19)	40(17)	48(25)	62(24)	55(17)
Functional scales									
PF	80(21)	85(17)	77(23)	87(14)	82(19)	67(20)	61(29)	82(21)	72(23)
RF	73(30)	84(20)	66(32)	76(29)	75(26)	57(30)	46(36)	74(30)	67(31)
EF	78(23)	84(22)	74(23)	81(18)	67(26)	75(22)	59(30)	79(24)	71(20)
CF	81(22)	90(17)	75(22)	83(17)	79(20)	60(24)	67(22)	83(21)	70(22)
SF	80(25)	89(23)	75(26)	85(26)	67(27)	69(23)	61(26)	81(26)	74(24)
Symptom scales									
Fatigue	23(20)	16(16)	28(21)	21(19)	29(22)	32(17)	40(21)	22(20)	31(19)
NV	8(20)	5(16)	10(22)	1.3(4.7)	15(29)	12(21)	18(31)	9(21)	7(15)
Pain	27(23)	20(19)	31(24)	20(17)	35(22)	41(22)	43(27)	26(23)	31(21)
Dyspnoea	16(25)	13(25)	17(25)	15(27)	15(25)	16(20)	36(26)	16(26)	15(22)
Insomnia	26(32)	20(28)	30(34)	19(29)	28(30)	38(37)	39(27)	25(33)	31(29)
AL	24(31)	15(23)	30(34)	22(32)	26(36)	44(33)	31(30)	23(32)	30(29)
Constipation	14(26)	8(15)	17(29)	11(27)	18(29)	28(32)	14(30)	14(26)	17(26)
Diarrhoea	11(24)	3(10)	17(28)	11(27)	18(29)	28(32)	14(30)	10(23)	17(26)
FD	32(34)	24(35)	36(33)	25(34)	45(35)	47(32)	47(30)	31(34)	33(34)

Mean (SD)- all given values are mean and standard deviation(SD) for QLQC-30 scores (the QLQC-30 scores are from 0–100, higher the score better the quality of life); VAS- visual analogue scale; Stage I -The cancer is less than 2 cm in size, and has not spread to lymph nodes in the area; Stage 2 - The cancer is more than 2 cm in size, but less than 4 cm, and has not spread to lymph nodes in the area; Stage 3 - The cancer is bigger than 4 cm but has not spread to any lymph nodes or other parts of the body; Stage 4 - The cancer is either greater than 4 cm in largest diameter or is of any size but has metastasised to regional lymph nodes; *GHS* global health status, *PF* physical functioning, *RF* role functioning, *EF* emotional functioning, *CF* social functioning, *NV* nausea and vomiting, *AL* appetite loss, *FD* financial difficulties

### Utility scores

**Fig. 1:**
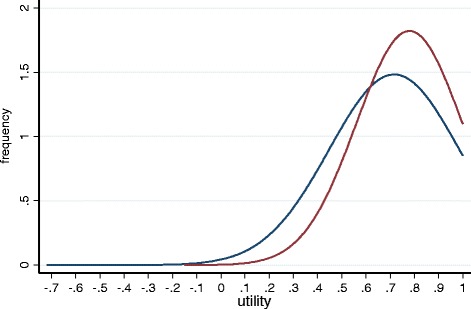
Distribution of the EQ-5D and the EORTC-8D utility scores. red: EORTC-8D distribution; blue = EQ-5D-3 L distribution

Figure 1 describes the distribution of the utility scores for both MAUIs. The distribution of both the EQ-5D-3 L and the EORTC-8D utility values were skewed to the right. Of the two, the EQ-5D-3 L shows more skewness and its distribution has more outliers. The lowest utility from the EQ-5D-3 L was −0.72 compared to −0.15 of the EORTC-8D.

### Psychometric properties of the two measures

#### Discrimination

##### Frequency

Patients were divided into five clinical severity classes for the four stages of cancer plus OPMD. Table 3 shows the mean and median utility scores and effect size for each class. All EORTC-8D mean utility scores were higher than the EQ-5D-3 L utility scores. Mean utility values reduced with increasing severity across both instruments. The EORTC-8D value for OPMD was the highest (0.9) mean score. For both instruments, utility differences across oral cancer severity classes were significant (*p* < 0.05, Kruskal-Wallis test) displaying good discriminating properties. Table 3 shows effect sizes for adjacent severity classes. As an example, 0.025 is the effect size for the mean utility difference observed between OPMD and cancer stage 1 for the EQ-5D-3 L. Except in one case, the effect sizes observed are higher for the EQ-5D-3 L than the EORTC-8D, suggesting better discrimination for the EQ-5D-3 L instrument.

**Table 3:** Discrimination between severity groups of oral cancer

MAUI	Cancer severity	Mean (SD)	Median	Effect size
EQ-5D-3 L				
	OPMD	0.78 (0.19)	0.79	
	Stage 1	0.76 (0.17)	0.78	0.025
	Stage 2	0.69 (0.28)	0.77	0.092
	Stage 3	0.57 (0.22)	0.56	0.174
	Stage 4	0.48 (0.46)	0.59	0.158
EORTC-8D				
	OPMD	0.84 (0.17)	0.90	
	Stage 1	0.83 (0.14)	0.85	0.011
	Stage 2	0.73 (0.23)	0.78	0.120
	Stage 3	0.65 (0.21)	0.63	0.109
	Stage 4	0.61 (0.32)	0.72	0.061

Stage 1 -The cancer is less than 2 cm in size, and has not spread to lymph nodes in the area; Stage 2 - The cancer is more than 2 cm in size, but less than 4 cm, and has not spread to lymph nodes in the area; Stage 3 - The cancer is bigger than 4 cm but has not spread to any lymph nodes or other parts of the body; Stage 4- The cancer is either greater than 4 cm in largest diameter or is of any size but has metastasised to regional lymph nodes; SD = standard deviation; Kruskal-Wallis test was used to determine the significance of difference between severity classes, *p* < 0.05; Wilcoxon matched paired rank test was used to test the significance between utility scores for each severity class, *p* < 0.05; MAUI = multi attribute utility instrument

### Responsiveness

Tables 4 and 5 show the distribution of the sample by the dimensions of the two MAUIs. A large number of people reported full health (no problems) within the EQ-5D-3 L. The related dimensions of mobility (EQ-5D-3 L) and physical functioning (EORTC-8D) recorded substantial differences. Percentage of patients who recorded no problems for mobility (EQ-5D-3 L) and physical functioning (EORTC-8D) dimensions were 76.2 % and 49 % respectively. The self-care dimension of the EQ-5D-3 L, which is also related to physical functioning, recorded 86 % of patients reporting they had no problems compared with 49 % reporting no problems with physical functioning in the EORTC-8D. However, both pain/discomfort and anxiety/depression dimensions of the EQ-5D-3 L had a similar percentage of patients reporting no problems compared with pain and emotional functioning dimensions of the EORTC-8D.

**Table 4:** Distribution of the EQ-5D-3 L dimensions

	Mobility	Self-care	Usual activities	Pain/discomfort	Anxiety /depression
No problem	115(76.2)	130(86.1)	107(70.9)	63(41.7)	91(60.3)
Some problem	34(22.5)	19(12.6)	41(27.1)	79(52.3)	56(37.1)
Extreme problem	2(1.3)	2(1.3)	3(2.0)	9(6)	4(2.7)

The numbers indicate the number of patients and percentage in each level of the five dimensions

**Table 5:** Distribution of the EORTC-8D dimensions

	Physical functioning	Role functioning	Pain	Emotional functioning	Social functioning	Fatigue	Nausea	Constipation and diarrhoea
Level 1	71(49)	75(50)	69(46)	93(62)	86(57)	106(70)	121(80)	105(70)
Level 2	48(32)	45(30)	58(39)	43(28)	51(34)	36(24)	19(13)	32(21)
Level 3	19(13)	18(12)	18(12)	8(5)	8(5)	6(4)	4(3)	8(5)
Level 4	6(4)	12(8)	5(3)	7(5)	6(4)	3(2)	7(5)	6(4)
Level 5	4(3)	-	-	-	-	-	-	-

The numbers indicate number of patients (and percentage) in each level of eight dimensions of the EORTC-8D

Few severe levels were reported by patients in both MAUIs, indicating floor effects. In comparison, the EORTC-8D had higher floor effects than the EQ-5D-3 L. This indicates that in severe conditions the EORTC-8D would be less responsive than the EQ-5D-3 L, but more responsive in mild conditions.

### Agreement

**Fig. 2:**
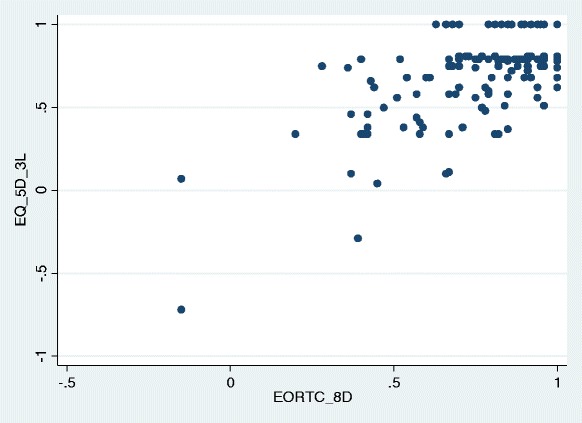
Correlation between EQ-5D-3 L and EORTC-8D utility scores

Figure 2 and Table 6 report the correlations between the two instruments. There is good agreement between the two utility scores for the better health states, but disagreement for severe health conditions (*r* = 0.69; *P* < 0.0001). Table 6 presents the correlation between EQ-5D-3 L and EORTC-8D dimensions, with those in bold for related dimensions. All correlations between related dimensions are significant (*p* < 0.05). The highest correlation (0.548) was observed between the mobility and physical function dimensions. The other related dimensions reported low correlations (*p* > 0.400). The dimensions fatigue, nausea and constipation/diarrhoea of the EORTC-8D did not have any related dimension with the EQ-5D-3 L.

**Table 6:** Correlation by dimensions

	Mobility	Self-Care	Usual activities	Pain/discomfort	Anxiety/depression
Physical functioning	**0.548**	**0.404**	0.475	0.385	0.335
Role functioning	0.309	0.268	**0.395**	0.159	0.399
Pain	0.384	0.322	0.393	**0.398**	0.359
Emotional functioning	0.303	0.291	0.282	0.163	**0.444**
Social functioning	0.364	0.241	**0.299**	0.297	0.442
Fatigue	0.209	0.212	0.204	0.313	0.236
Nausea	0.268	0.242	0.143	0.290	0.306
Constipation and diarrhoea	0.230	0.152	0.093	0.286	0.143

The bold figures show the correlation between similar dimnesions of the two instruments

### Difference across MAUIs

Table 3 shows there are differences between the EQ-5D-3 L and the EORTC-8D utility scores for the same health condition experienced by patients. There are significant differences between instruments for OPMD, oral cancer stage 1 and stage 3 (P < 0.05). The low correlation between related dimensions (Table 6) suggests the two MAUIs capture different aspects of health in these patients.

## Discussion

This is the first study to examine the difference between utility values estimated by the EQ-5D-3 L and the EORTC-8D for health states described by a sample of OPMD and oral cancer patients. This is also the first study to produce a comparison of generic and disease specific preference based measures from a low/middle income country [27, 28], and is robust in that it used country-specific algorithms for both the EQ-5D-3 L and the EORTC-8D [29].

It is noteworthy that, similar to Rowen et al. [10], our analysis found the EQ-5D-3 L to have higher discrimination across cancer severity groups than the EORTC-8D, the cancer specific preference based measure. A possible explanation for the finding that the EORTC-8D lacked discrimination between individuals with different severities of disease could lie in the algorithm used to convert QLQC-30 values to EORTC-8D utility scores. This algorithm was based on a regression model which estimated the utility scores [21]. The preferred model in the regression analysis did not consider all the levels in the EORTC-8D as they were sometimes not significant or inconsistent [21]. Another reason could be the different time periods which patients are asked to consider in the two instruments: the EQ-5D-3 L asks respondents to consider their current health, whereas QLQC-30 asks them to consider the past week.

Rowen et al. previously compared the EORTC-8D and the EQ-5D-3 L utility values using a group of patients with multiple myeloma in the UK [10]. They used the Karnofsky performance scale to determine the severity levels of their patients, whereas we used the clinical severity groups described [10]. There is a clear distinction between the clinical definition of an OPMD, of an overt oral malignancy, and the four stages of oral cancer: this enabled us to group our patients without involving extra scales. We observed that participants with a greater tumour size reported lower quality of life. As reporting QoL of the patients was not the main purpose of this paper, we only used average of functional and symptom scales from the QLQC-30 data. However, averaging the functional and symptom scales did not reduce the ability of the scales to discriminate QoL by the severity levels. Patients who were waiting for treatment had a lower QoL, perhaps explained by high anxiety or severe discomfort. In our analysis, the EORTC-8D values are very similar to the average of functional scores but higher than the global health scores.

Our analysis contributes to the discussion of need for a disease specific preference based measure in cancer research. We found significant differences between the EQ-5D-3 L and the EORTC-8D utility values in most of the severity groups of OPMD and oral cancer. In addition, we found the EORTC-8D utility values to be significantly higher than the EQ-5D-3 L utility values for a given disease state. We also found the EQ-5D-3 L was more discriminating than the EORTC-8D across the majority of severity groups. In severe health conditions the EQ-5D-3 L is more discriminating that the EORTC-8D due to its larger effect size. This possibly indicates the ability of EQ-5D-3 L to discriminate severe cancer health states as well as, or better than, the present cancer specific method. This poses serious challenges to the conventional understanding that disease specific instruments are likely to have greater discrimination. The significant difference shown in discrimination was of practical importance as shown by the moderate effect sizes. Such differences could be pivotal in determining whether or not a particular health intervention is cost effective. The lower utility of the EQ-5D-3 L in severe health states would reflect higher utility gain in a health intervention where patients gain better health. Thus, it is quite possible the EQ-5D-3 L would show a higher outcome gain in QALYs than the EORTC-8D in an economic analysis.

Although our study did not have longitudinal data, so that responsiveness over a time period could not be determined, we have derived information on ceiling and floor effects of both MAUIs. The EQ-5D-3 L showed high ceiling effects with large percentages reported in full health. The influence of ceiling effects was less in the EORTC-8D. This indicates that EORTC-8D could be more responsive in mild health conditions than the EQ-5D-3 L. The higher number of levels in the EORTC-8D, as well as the fact that respondents rate the QLQC-30 instead of directly rating EORTC-8D, could also in part account for this.

There is low correlation between the dimensions of the two MAUIs. This suggests the dimensions of the two MAUIs capture different aspects of health. The EORTC-8D was capable of explaining different aspects of quality of life to EQ-5D-3 L. The dimensions of fatigue, nausea and constipation/diarrhoea of the EORTC-8D did not have any related dimensions with the EQ-5D-3 L, reinforcing this concept.

Our results are contrary to the current belief that disease specific instruments are more sensitive than generic MAUIs. However, inherent limitations in the development of the disease specific MAUI considered in this analysis could be a factor. The EORTC-8D was developed using QLQC30 data of only one cancer condition. It was not validated in other types of cancer. Further research revisiting the psychometric analysis which produced the original EORTC-8D classification system is recommended.

### Limitations

The cross-sectional nature of our data prevented examination of responsiveness of the utility values over time. We did not consider the time spent in a given health state. There is a possibility that remaining in a particular health state for a long time allows patients to adapt, and thus deliver higher utility scores. In the absence of longitudinal data, standardised response mean, the best method to measure responsiveness could not be calculated. Instead we used the floor and ceiling effects which can only indirectly imply potentially limited responsiveness. Additionally, as the EQ-5D-3 L was always applied ahead of the QLQC30 there is a potential ordering bias in our data. Ideally, half of the patients randomly selected should have received one questionnaire first and the other half the next questionnaire first.

## Conclusion

This is one of a few studies comparing a disease specific preference-based instrument with a generic instrument. Moreover, this is the first study to use OPMD and oral cancer, these being a major public health problem in the whole of South Asia. The EQ-5D-3 L and the EORTC-8D utility values were significantly different from each other. Whilst both preference-based measurements were able to discriminate between the severity stages of OPMD and oral cancer, the EQ-5D-3 L utility values were always lower than the EORTC-8D values for given cancer health states. Our findings suggest that whilst the disease specific EORTC-8D may be superior for valuing mild oral health states, the generic EQ-5D-3 L may be superior for valuing severe oral health states. Whilst the ceiling effect of the EQ-5D-3 L is well recognised, the suggestion that a generic instrument has superior discrimination to a disease specific instrument challenges conventional wisdom, and should be explored further. The observed differences could result in quite different outcomes when determining whether or not a health intervention was cost effective, and this could have important implications for policy decisions.
